# Individual and environmental factors associated for overweight in urban population of Brazil

**DOI:** 10.1186/1471-2458-13-988

**Published:** 2013-10-21

**Authors:** Larissa L Mendes, Helena Nogueira, Cristina Padez, Maria Ferrao, Gustavo Velasquez-Melendez

**Affiliations:** 1 Departamento de Nutrição, Instituto de Ciências Biológicas, Universidade Federal de Juiz de Fora, Juiz de Fora 36036-900, Brazil; 2 Department of Geography, University of Coimbra, Largo da Porta Férrea, Coimbra 3004-530, Portugal; 3 Department of Life Sciences, University of Coimbra, Coimbra 3004-530, Portugal; 4 Research Centre for Anthropology and Health, University of Coimbra, Coimbra 3004-530, Portugal; 5 Departamento de Enfermagem Materno-infantil em Saúde Pública, Escola de Enfermagem, Universidade Federal de Minas Gerais, Belo Horizonte 30130-100, Brazil

## Abstract

**Background:**

Obesity is a significant global public health problem and the main cause of many chronic diseases in both developed and developing countries. The increase in obesity in different populations worldwide cannot be explained solely by metabolic and genetic factors; environmental and social factors also have a strong association with obesity. Thus, it is believed that the current obesity epidemic is the result of a complex combination of genetic factors and an obesogenic environment .The purpose of this study was to evaluate individual variables and variables within the built and social environment for their potential association with overweight and obesity in an urban Brazilian population.

**Methods:**

Cross-sectional study was carried out in a sample of 3404 adults living in the urban area of the city. Information from the surveillance system for chronic diseases of Brazilian Ministry of Health was used and individual data was collected by telephone interviews. The database was geocoded using the Brazilian System of Postal Codes for participant residences. An updated, existing list based on the current addresses of supermarkets and hypermarkets in the city was used as an indicator variable of the availability and access to food. Georeferenced information on parks, public squares, places for practicing physical activity and the population density were also used to create data on the built environment. To characterize the social environment, we used the health vulnerability index (HVI) and georeferenced data for homicide locations.

**Results:**

The prevalence was 44% for overweight, poisson regression was used to create the final model. The environment variables that independently associated with overweight were the highest population density, very high health vulnerability index and the homicide rate adjusted for individuals variables. The results of the current study illustrate and confirm some important associations between individual and environmental variables and overweight in a representative sample of adults in the Brazilian urban context.

**Conclusions:**

The social environment variables relating to the socioeconomic deprivation of the neighborhood and the built environment variables relating to higher walkability were significantly associated with overweight and obesity in Belo Horizonte.

## Background

Obesity is a significant global public health problem and the main cause of many chronic diseases in both developed and developing countries [1-3]. Study conducted in Brazil showed that both male and female overweight individuals account for 50% of the population, while the prevalence of obesity is 12.5% for men and 16.9% for women [4].

Briefly, weight gain occurs when the energy balance is positive, that is, when the calories consumed are greater than the calories expended. However, the increase in obesity in different populations worldwide cannot be explained solely by metabolic and genetic factors; environmental and social factors also have a strong association with obesity [5]. Thus, it is believed that the current obesity epidemic is the result of a complex combination of genetic factors and an obesogenic environment [6,7].

The ecological model proposed by Swinburn *et al*. [7] that is used to analyze the causes of obesity considers the environment an important determinant of obesity, one that directly affects the dietary behavior and physical activity patterns of the individual and, therefore, their energy balance. In this context, the environment is divided into two parts, dimension (micro or macro) and type (physical, economic, political and sociocultural). The physical environment, also defined as the built environment, is the most studied dimension. It refers to the physical aspects of an environment built or modified by man and favors unhealthy behaviors within the community [8]. Thus, the built environment may limit healthy eating habits, either through limiting access to fresh fruits and vegetables [9] or by having a high density of fast food restaurants and convenience stores [10]; it may also create barriers that limit physical activity [11-14]. Moreover, the social environment can also affect healthy eating habits. Communities with higher socioeconomic deprivation have fewer opportunities to access healthy food [15,16] or to practice physical activity, either for leisure or for travel by foot or bicycle [17,18]. Thus, obesity-related policies and programs should encourage individual changes as well as changes to the society and the environment in which people live and work [19].

In Brazil, studies evaluating the physical and social environments related to obesity are still scarce, making it critical to investigate and identify potential obesogenic environments within Brazilian cities. The purpose of this study was to evaluate individual variables and variables within the built and social environment for their potential association with overweight and obesity in an urban Brazilian population.

## Methods

### Study population

Participants were selected from the Surveillance of Risk Factors for Chronic Diseases through Telephone Interview (VIGITEL), organized by the Ministry of Health and conducted annually in all Brazilian state capitals. For the present study, samples from the years 2008 and 2009 for the city of Belo Horizonte were used. The city of Belo Horizonte is the capital of the Minas Gerais State, is located in the southeastern region, has a total area of 331 Km^2^, population of 2.365.151 inhabitants and population density of 7.177 habitants Km^2^ according to the population census of the year 2010.

Sampling of participants was conducted in two steps: (1) random selection of households with landlines and (2) drawing of respondents 18 years of age or older. The VIGITEL system seeks to obtain random population samples of adults living in households with a landline from each Brazilian state capital. Sample weights for the individuals interviewed were later determined to correct potential biases resulting from the non-universal coverage of the telephone network. For the present study, 4.000 interviews were considered eligible, of which 3,661 interviews containing individual weight and height information were used. We also used data from a standardized questionnaire, delivered by phone interview, which collected self-referred information regarding sociodemographic characteristics, eating patterns, weight, height, physical activity and health-related variables. Of the original sample, 101 individuals (3.6%) were excluded due to a body mass index (BMI) of less than 18.5 kg/m^2^ and an additional 125 individuals were excluded due to errors in georeferencing. The final sample consisted of 3,404 individuals.

### Dependent variable

BMI was considered the dependent variable in this study and was categorized into two groups, normal (18 kg/m^2^ ≤ BMI < 25 kg/m^2^) and overweight (BMI ≥ 25 kg/m^2^), according to the values proposed by the World Health Organization [3]. Data on weight and height were self-reported. These data have been widely used in epidemiological studies [20] and have been previously validated for Brazilian adults. High correlation coefficients were found when the measurements were compared to the corresponding self-referred values and showed good results when analyzed for sensitivity and specificity [21].

### Independent variables

The independent variables used in the study were based on a review of the literature and were selected from the VIGITEL database and the available geocoded data. Individual variables were divided into three categories: sociodemographic, lifestyle and health. Some variables relating to diet and physical activity were also validated in previous studies [22,23].

### Characterization of geographic data

To verify and analyze the spatial distribution of variables, the VIGITEL database was geocoded using the Brazilian System of Postal Codes (CEP) for participant residences [24]. To characterize the built and social environment, a geocoded base was developed that incorporated the individual data for each participant. An updated, existing list based on the current addresses of supermarkets and hypermarkets in the city was used as an indicator variable of the availability and access to food. Georeferenced information on parks, public squares and places for practicing physical activity and the population density were also used to create data on the built environment. These data were provided by the Computer and Information Company of Belo Horizonte.

To characterize the social environment, we used the health vulnerability index (HVI), which highlights the inequalities in the epidemiological profile of different social groups. This measurement combines different socioeconomic and environmental variables in a single synthetic indicator. The HVI can be understood as the result of combining both socioeconomic and demographic dimensions. Census tracts and their residents are classified into groups according to social vulnerability. In addition, georeferenced data for homicide locations were used to map homicide rates by census tracts. These data were provided by the Integrated Information Center for Social Defense of the Military Police of Minas Gerais. The median household income per census tract was obtained from the databases of the Brazilian Institute of Geography and Statistics was used as a social environment variable.

### Data analysis

#### Spatial data analysis

A spatial analysis using the Geographic Information System (GIS) was first performed to stratify the geocoded data into layers of information, to isolate the spatial relationships and to develop data maps. The spatial information was then crosschecked and translated into variables to be analyzed. These procedures were performed using the MapInfo program version 10.5. The information for the exact location (latitude and longitude) based on the census tract was used to analyze the spatial distribution of cases. At this level, it is appropriate to consider the impact of social relationships, characteristics of the built environment and services and amenities on the health of individuals [25,26].

#### Statistical analysis

The survey module in the STATA 12 program was used for the analysis of individual and environmental data. Various aspects of the complex sampling design are considered in the analysis as post-stratification weights and are assigned to the interviewed participants to allow for the partial correction of bias arising from the non-universal coverage of the phone network [27]. Prevalence ratios (PRs) were used as a measure of association in the bivariate analysis; the ratios were calculated using Poisson regression analysis with robust variance because this was a cross-sectional design in which the searched outcome was very frequent [28]. Statistical differences were evaluated according to the likelihood ratio (LR). Initially, the sample description was presented according to the participants’ BMI in addition to the bivariate associations with the variables relating to the built and social environments. The bivariate analysis was initially performed to identify which explanatory variables had the greatest influence on variations in overweight. Since the respondents were not sampled by a multi-stage sampling method (i.e., they were not geographically clustered), we report the results estimated by single level analysis (we confirmed that using multilevel modelling did not alter our findings). Two multiple regression models were fitted to determine the association of overweight with individual and environmental variables.

### Ethical issues

The present study was approved by the National Committee for Ethics in Human Research, part of the Ministry of Health, and by the Ethics Committee of the Federal University of Minas Gerais, according to protocol number 552/08.

## Results

The final sample consisted of 3.404 individuals of whom 49.9% were male and 50.1% were female. The average age of the participants was 39.7 years, 44,0% of individuals were overweight (31.60% with BMI ≥ 25 kg/m^2^; 12.40% with BMI ≥ 30 kg/m^2^). Table 1 shows the sociodemographic characteristics of the sample. When comparing individuals with normal BMI (BMI < 25 kg/m^2^) to overweight individuals (BMI ≥ 25 kg/m^2^), significant differences were observed in sex, age distribution, marital status and education.

**Table 1:** Sociodemographic characteristics for all participants by BMI

**Variables**	**Total**		**BMI < 25 kg/m**^**2**^		**BMI ≥ 25 kg/m**^**2**^	
	**%**	**95 CI%**	**%**	**95 CI%**	**%**	**95 CI%**
Sex*						
Males	50.10	47.53–52.74	46.90	43.14–50.64	53.10	49.40–56.90
Females	49.90	47.26–52.47	41.10	37.50–44.80	58.90	55.20–62.50
Age (years)*						
18–24	19.40	16.80–22.50	22.50	14.50–33.20	77.50	66.8–85.50
25–34	23.50	21.30–26.00	41.20	35.40–47.30	58.80	52.70–64.60
35–44	22.80	21.00–24.70	48.10	43.90–52.40	51.90	47.60–52.10
45–54	15.70	14.20–17.00	53.20	48.70–57.60	46.80	42.40–51.30
55–64	10.00	9.00–11.20	60.70	55.50–65.60	39.30	34.40–44.50
≥65	8.60	7.60–9.50	53.00	47.70–58.20	47.00	41.80–53.00
**Skin Color**						
White	34.30	32.15–36.53	42.40	39.17–45	57.60	54.40–60.80
Non White	65.70	63.47–67.85	44.90	41.30–48.50	55.10	51.50–58.70
Marital Status*						
Single	43.00	40.20–45.70	34.40	29.60–39.70	65.60	60.40–70.40
Married	47.60	45.10–50.20	50.50	47.60–53.40	49.50	46.60–52.40
Others(seperated/divorced/widowed)	9.40	8.30–10.70	54.60	48.40–60.60	45.40	39.40–51.60
Education (years)*						
≤4	17.00	14.60–19.76	50.80	42.20–59.40	49.20	40.60–57.80
5–8	30.80	28.15–33.60	46.50	40.90–52.20	53.50	47.80–59.10
9–11	31.60	29.58–33.66	41.60	38.60–44.70	58.40	55.30–61.40
≥12	20.60	19.07–22.20	37.50	34.20–41.00	62.50	58.90–65.90

Notes: ^a^Percentages weighted to adjust the sociodemographic distribution of the VIGITEL sample to the distribution of the population 18 years and older from the city according to the 2000 Census. *p < 0.05 (Chi-square). N = 3.405.

Table 2 presents the raw association of variables related to the built and social environment with overweight. For the social environment variables, a lower prevalence of overweight and obesity was found in the fourth quartile of population density while a higher prevalence was found in the higher HVI category (p < 0.05).

**Table 2:** Prevalence ratios and 95% confidence intervals for overweight/obesity and variables for the built and social environment in the census tracts

**Environment variables**	**%**	**PR**	**PR 95% CI**
**Built environment**			
**Presence of supermarkets**			
No	44.10	1.00	-
Yes	42.60	0.97	0.78–1.20
**Presence of fruit and vegetables markets**			
No	44.50	1.00	-
Yes	34.70	0.78	0.57–1.06
**Parks/public squares/places for practicing physical activity**			
No	44.00	1.00	-
Yes	43.90	0.99	0.72–1.37
**Population density**			
1st	49.40	1.00	-
2nd /3rd/ 4th quartil	42.30	0.85	0.74–0.98
**Social environment**			
**HVI**			
Low	43.70	1.00	-
Medium	43.80	1.01	0.86–1.16
High	42.80	0.98	0.82–1.16
Very High*	58.00	1.33	1.04–1.69
Homicide rate (100s)*	46.10	1.19	0.85–1.65
**Household income (real)**			
1st quartile	44.60	1.00	-
2nd /3^rd^/4th quartile	43.80	0.98	0.86–1.11

Notes: HVI: Health Vulnerability Index. *p ≤ 0.05.

We used an adjusted Poisson regression model in a single level, as shown in Table 3.

**Table 3:** Final Poisson regression model with overweighthobesity as a response variable

**Variables**	Model 1*		Model 2 **	
	**PR**	**PR 95% CI**	**PR**	**95% CI**
**Built environment**				
**Population density**			-	-
1st	1	-		
2nd /3rd/ 4th quartil	0.86	0.75–0.98		
**Social environment**				
**HVI**				
Low	1	-	1	-
Medium	1.02	0.88–1.17	1.07	0.92–1.24
Hight	0.99	0.85–1.16	1.02	0.86–1.22
Very hight	1.32	1.04–1.67	1.37	1.05–1.80
**Homicide rate (1000s)**	-	-	1.45	1.02–2.05

*Model 1 adjusted for sex.

**Models 2 adjusted for sex, age and commute to work by food or bicycle.

Notes: HVI: Health Vulnerability Index.

In Model 1, the environment variables that independently associated with overweight were the highest population density (PR = 0.86) and the very high HVI (PR = 1.32) adjusted for sex and habit of watching TV every day of the week. In Model 2, the environment variables that independently associated with overweight the very high HVI (PR = 1.37) and the homicide rate (RP= 1.45) adjusted for sex, age and the commute to work by food and bicycle. We tested the interactions between the variables in the final model, but they were not significant.

## Discussion and conclusions

The results of the current study illustrate and confirm some important associations between individual and environmental variables and overweight in a representative sample of adults in the Brazilian urban context. The relationship between the environment (built and social) and obesity has long been studied in developed countries [7,29,30], but in Brazil, this type of research is relatively recent [31,32].

In this study, living in neighborhoods with a high population density was inversely associated with a prevalence of overweight. According to Frank *et al*. [33], neighborhoods with high population density often exhibit greater mixed land use and greater street connectivity; consequently, they promote higher levels of foot traffic (walkability), which may be related to lower BMI.

Identical results were found in a study conducted in New York. People who lived in census tracts with higher population density had significantly lower BMIs than those who lived in areas with low population density. This study also found an inverse association between BMI and other variables of the built environment such as mixed land use, the number of bus stops and subway stations and the number of street intersections [25].

Another study using data from the Behavioral Risk Factor Surveillance System (BRFSS) in Massachusetts found that after adjusting by individual variables, population density was inversely associated with obesity. This study suggests that the highest population density was associated with greater walkability, indicating that cars were used less frequently, thus increasing physical activity [34].

When exploring the contributions of the social environment to the prevalence of overweight, it was found that communities with a high health vulnerability index (HVI) presented the highest prevalence of overweight. The relationship between higher rates of overweight and obesity in populations with poor socioeconomic conditions is well established in developed and many developing countries [35,36]. Furthermore, studies show that individuals living in socially disadvantaged neighborhoods, which tend to be characterized by insecure and dangerous environments, present a higher risk of having unhealthy behaviors [37-40] with respect to diet [41,42] or physical activity [43-46].

A longitudinal study conducted in England found that women living in economically disadvantaged neighborhoods had higher BMI and greater weight gain compared to women in more affluent neighborhoods. The study also found that over a period of ten years, women from disadvantaged neighborhoods gained one kilogram more than women from neighborhoods that were more affluent. No associations were found for men between socioeconomic status of the neighborhood and BMI [47].

Identical results were found in other studies. One study conducted in the United States revealed that adults who lived in neighborhoods with low socioeconomic conditions presented mean BMI values that were higher than the BMI values of adults living in neighborhoods with better socioeconomic conditions [16]. Another study conducted in the Netherlands found that the odds ratio for the occurrence of overweight significantly increases with deprivation in the neighborhood [46].

A number of findings suggest that the socioeconomic environment of the neighborhood is related to the prevalence of overweight and obesity through mediators related to the built environment, such as access to healthy [48,49] and unhealthy food stores [15,50]. Thus, economically favored neighborhoods tend to have a higher density and a greater number of supermarkets and grocery stores that are associated with a greater consumption of healthy foods and lower rates of overweight and obesity [51].

In the urban context of Brazil, only one study, conducted in São Paulo, showed that there was a higher density of markets specializing in fruits and vegetables in the city's wealthiest areas. However, no associations between the availability of healthy foods in the neighborhood and overweight were observed [31]. Similarly, the present study showed no associations with overweight when the contributions of the built environment based on the availability of healthy foods in the neighborhood were investigated.

However, the food environment (food availability and access) alone does not explain the association between neighborhood deprivation and obesity. Other aspects of the built environment related to physical inactivity and/or physical activity may contribute this association. Several studies have found that economically disadvantaged neighborhoods are characterized by offering fewer opportunities for physically activity [52-54].

Also in relation to the social environment, an association was found between the highest homicide rate in the census tract and the highest prevalence of overweight. Previous findings suggest that higher rates of obesity are found in neighborhoods characterized by social disorder [42,55]. This association may be mediated by restrictions on physical activity as lower rates of walking and other physical activities or outdoors due to fear [44,56,57]. A study with adults found that the increased perception of safety for the crime was significantly associated with lower BMI values. The authors suggest that the use of objective measures of security, such as homicide rates and crimes in the neighborhood, may be more related to BMI than measurements based on the perception of individuals [58]. Another study found similar results for the perception of security, since individuals who perceived their neighborhoods as unsafe had higher BMI than those who perceived the neighborhood as safe. These associations remained after adjustment for individual, sociodemographic variables and the socioeconomic status of the neighborhood [40].

The present study does have certain limitations. First, despite the use of a causal model, the cross-sectional design does not allow us to draw such conclusions. A second limitation is inherent in the proposed methodology because it uses health surveillance systems. Although it is a practical methodology, it depends on self-referred measurements reported by phone, which may include potential biases depending on the variable being assessed. Third, we used secondary data from governmental and commercial sources to describe the environmental characteristics (built and social), but these may also be subject to inaccuracies. Other important limitation of this study is the fact of the walkability was not measured and this is an important aspect of the obesogenic environments. Finally, we recognize that there is a limitation with respect to sample selection, as only people living in households with a landline participated in this study. However, sample weights were taken into consideration when analyzing the data to adjust for the sociodemographic composition of the population.

In conclusion, the social environment variables relating to the socioeconomic deprivation of the neighborhood and the built environment variables relating to higher walkability were significantly associated with overweight in Belo Horizonte, Brazil. These initial findings require further investigation to account for the realities of other Brazilian cities. The inclusion of other cities in future research will facilitate the understanding of the role of environments (built and social) in the current obesity epidemic, thus allowing for the development of useful tools able to generate effective strategies for the prevention of adult obesity in the Brazilian context.

## Competing interests

The authors declare that they have no competing interests.

## Authors’ contributions

GVM and LM assisted with the study coordination, data management, carried out the statistical analysis and drafted the manuscript. CP, HN and MF helped with the statistical analysis and helped to draft and revise the manuscript. All authors read and approved the final manuscript.

## Pre-publication history

The pre-publication history for this paper can be accessed here:

http://www.biomedcentral.com/1471-2458/13/988/prepub
